# Malposition of a Peripherally Inserted Central Catheter Into the Internal Vertebral Venous Plexus in a Neonate With Central Nervous System Malformations

**DOI:** 10.7759/cureus.105148

**Published:** 2026-03-13

**Authors:** Masatoshi Oshima, Katsunori Tanaka, Kazunori Mori, Tomoaki Kitamura, Yoshihiro Maruo

**Affiliations:** 1 Pediatric Medicine, Shiga University of Medical Science, Otsu, JPN; 2 Neurological Surgery, Shiga University of Medical Science, Otsu, JPN

**Keywords:** computed tomography, internal vertebral venous plexus, malposition, myelomeningocele, peripherally inserted central venous catheter

## Abstract

Peripherally inserted central catheters (PICCs) are widely used in neonatal intensive care units (NICUs); however, malposition into the paravertebral venous system is a rare but potentially life-threatening complication. Migration of a PICC into the internal vertebral venous plexus is particularly uncommon, and direct imaging confirmation of this pathway has rarely been reported.

We report a term neonate with myelomeningocele, Chiari II malformation, and hydrocephalus in whom a PICC inserted via the left great saphenous vein migrated from the ascending lumbar vein into the internal vertebral venous plexus. Chest-abdominal radiography was obtained for catheter position confirmation, and computed tomography (CT) imaging was subsequently performed for preoperative assessment of the myelomeningocele. CT imaging incidentally but definitively demonstrated the continuous migration pathway of the catheter into the spinal canal. The catheter was removed before the initiation of hyperosmolar infusions, and no complications occurred.

Malposition of PICCs into the internal vertebral venous plexus poses a substantial risk of severe neurological and potentially fatal complications. In the present case, CT imaging enabled precise anatomical confirmation of catheter migration that could not be achieved by plain radiography alone.

To the best of our knowledge, this is the first neonatal case in which migration of a PICC from the ascending lumbar vein into the internal vertebral venous plexus was precisely demonstrated using CT imaging. This case underscores the importance of careful catheter position assessment and highlights the conditional but critical role of CT in accurately diagnosing dangerous PICC malposition.

## Introduction

Peripherally inserted central catheters (PICCs) are widely used in neonatal intensive care units (NICUs) for long-term intravenous therapy and parenteral nutrition. Although generally considered safe, PICC-related complications include phlebitis, catheter occlusion, catheter-related bloodstream infection, and catheter malposition. Several studies have demonstrated that these complications are associated with the insertion site and catheter tip position, and accurate confirmation of catheter tip location before use is therefore strongly recommended, particularly in neonates [1,2].

When PICCs are inserted via lower extremity veins in neonates, the ideal tip position is within the inferior vena cava [1]. However, malposition into the paravertebral venous system has been reported and may lead to serious complications [3]. The paravertebral venous system includes the ascending lumbar vein and the internal vertebral venous plexus, which communicate through intervertebral veins and form a valveless venous network adjacent to the vertebral column. Most previously reported cases of PICC malposition involve the ascending lumbar vein, typically inferred from plain radiographic findings [3]. In contrast, migration into the internal vertebral venous plexus, which is located within the spinal canal and anatomically closer to the central nervous system, appears to be uncommon and may carry a higher risk of neurological complications.

A previous neonatal case described suspected catheter migration into the internal vertebral venous plexus following PICC insertion via the left great saphenous vein, based on radiographic findings and the development of neurological symptoms such as segmental myoclonus [4]. However, in that report, the catheter position was inferred from radiography alone, and precise anatomical confirmation of the catheter pathway was not demonstrated using cross-sectional imaging. Therefore, detailed visualization of catheter migration into the internal vertebral venous plexus remains limited in the neonatal literature.

Myelomeningocele is frequently associated with Chiari II malformation and hydrocephalus and is characterized by congenital abnormalities of the spine and surrounding structures [5,6]. The potential influence of these anatomical abnormalities on the trajectory of PICCs inserted via lower extremity veins has not been sufficiently investigated.

Here, we report a term neonate with myelomeningocele, Chiari II malformation, and hydrocephalus in whom a PICC inserted via the left great saphenous vein migrated through the left ascending lumbar vein into the internal vertebral venous plexus.

The aberrant catheter pathway was definitively confirmed by computed tomography (CT), with radiographic findings consistent with malposition. We present this case with a review of the relevant literature because of its educational value and clinical implications.

## Case presentation

**Figure 1:**
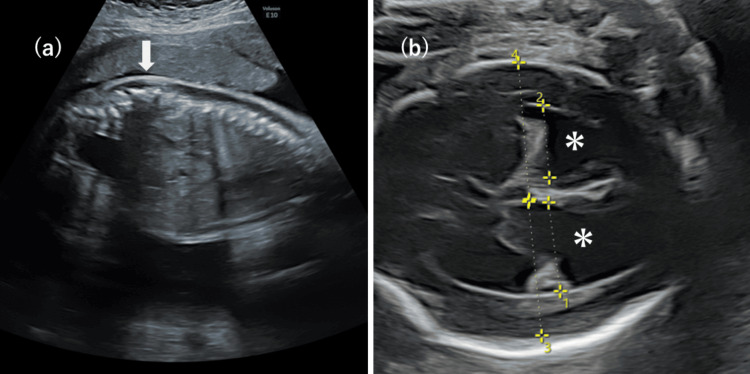
Prenatal ultrasonographic findings demonstrating central nervous system malformations Prenatal ultrasonography shows a lumbosacral myelomeningocele (arrow) and ventriculomegaly consistent with hydrocephalus (asterisk).

Prenatal ultrasonography revealed Chiari II malformation, hydrocephalus, and myelomeningocele (Figure 1a, 1b). The patient was delivered by scheduled cesarean section at 37 weeks and five days of gestation. Birth weight was 2,688 g (0.0 SD), length 47 cm (-0.3 SD), and head circumference 34.5 cm (+1.3 SD). Apgar scores were 8 at one minute and 8 at five minutes. The patient was male.

**Figure 2:**
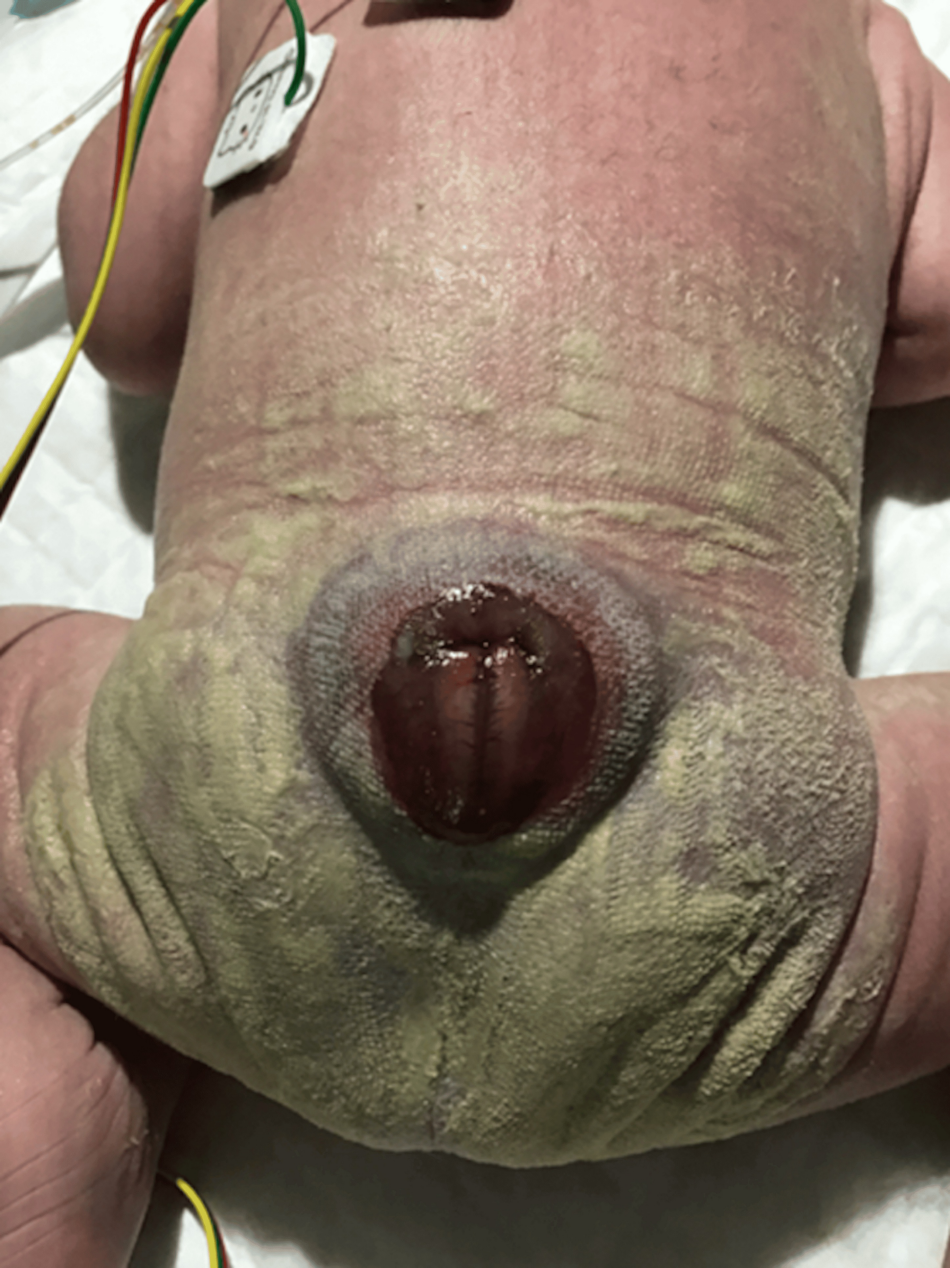
Clinical photograph of the dorsal lesion The image shows a lumbosacral myelomeningocele.

A myelomeningocele lesion was observed on the back (Figure 2). Resuscitation was performed with care to avoid pressure on the lesion, and the infant was managed in the prone position after admission to the NICU. No cerebrospinal fluid leakage was observed. Spontaneous movements of all extremities were active, muscle tone was mildly decreased, and plantar grasp reflexes were present bilaterally. Weak plantar flexion of the ankles was noted.

On day 0, a peripherally inserted central catheter (PICC) was inserted via the left great saphenous vein. The catheter was advanced without resistance, and blood return was confirmed. A 27-gauge catheter (outer diameter: 0.43 mm, total length: 20 cm) was used, and the catheter was inserted to its full length. On the same day, chest-abdominal radiography was obtained to confirm the catheter position. Before formal interpretation of the radiographic findings was completed, non-contrast computed tomography (CT) was performed primarily for preoperative assessment of the myelomeningocele. The dose-length product (DLP) for the CT examination was 36.7 mGy·cm.

**Figure 3:**
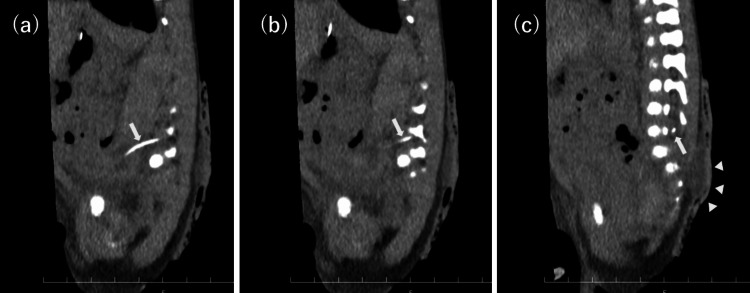
Sagittal CT image demonstrating posterior protrusion of the thecal sac at the sacral level Sagittal CT shows posterior protrusion of the thecal sac consistent with spina bifida at the sacral level (arrowhead). The peripherally inserted central catheter is also visible within the thecal sac (arrow). CT: computed tomography

**Figure 4:**
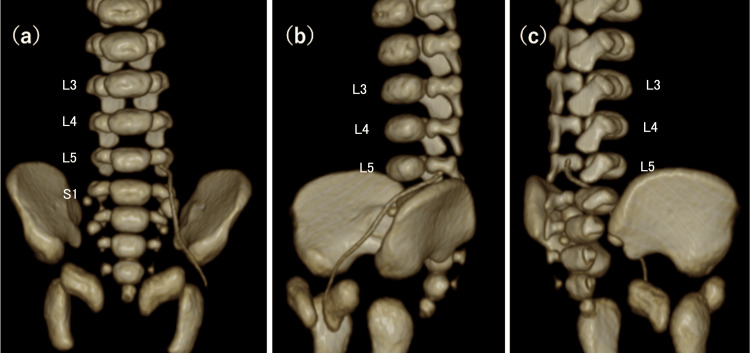
Three-dimensional reconstructed CT These images show the catheter passing from the left ascending lumbar vein through the intervertebral vein into the internal vertebral venous plexus, with the catheter tip located within the spinal canal. Vertebral levels (L3, L4, L5, and S1) are labeled on this image. (a) Anterior view. (b) Left anterolateral view. (c) Right posterolateral view. CT: computed tomography

**Figure 5:**
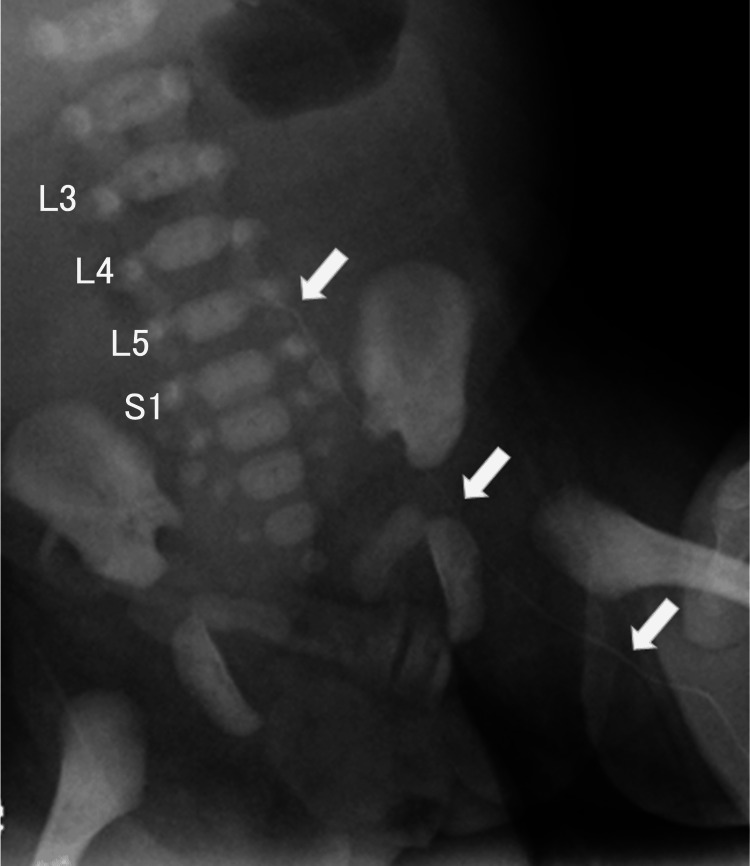
Chest-abdominal radiograph showing an abnormal course of the catheter The catheter (arrow) ascends along the left side of the vertebral column and curves medially at the level of the fifth lumbar vertebra. Vertebral levels (L3, L4, L5, and S1) are labeled on this image.

CT imaging revealed spina bifida with posterior protrusion of the thecal sac at the sacral level and incidentally allowed precise evaluation of the catheter position (Figure 3a-3c), demonstrating that the catheter had migrated from the left ascending lumbar vein through the intervertebral vein into the internal vertebral venous plexus, with the catheter tip located within the spinal canal (Figure 4a-4c). Subsequent review of the chest-abdominal radiograph showed that the catheter ascended along the left side of the vertebral column and curved medially at approximately the level of the fifth lumbar vertebra, findings consistent with the abnormal catheter course identified on CT (Figure 5). CT imaging allowed precise three-dimensional localization of the catheter tip relative to the vertebral canal and paravertebral venous system. On plain radiography, the catheter trajectory overlapped with the vertebral column and pelvic structures, which can obscure abnormal posterior or paravertebral courses. CT, therefore, enabled clear identification of catheter migration into the internal vertebral venous plexus.

Blood return, rather than cerebrospinal fluid, was confirmed from the catheter. The PICC was carefully removed before the initiation of hyperosmolar infusions such as parenteral nutrition. No neurological deterioration or other complications were observed following catheter removal.

On day 1, external ventricular drainage and surgical repair of the myelomeningocele were performed under general anesthesia. Due to persistent hydrocephalus, a ventriculoperitoneal shunt was placed on day 17. The clinical course was favorable, and the patient was discharged on day 33 without complications.

The clinical timeline, including PICC insertion, imaging studies, and catheter removal, is summarized in Table 1.

**Table 1:** Clinical timeline of the present case CT: computed tomography, PICC: peripherally inserted central catheter

Time after birth	Clinical event
~1 hour	PICC inserted via the left great saphenous vein
Shortly after insertion	Radiography performed for catheter position confirmation
Same day	Non-contrast CT obtained for preoperative evaluation; catheter migration into the internal vertebral
Several hours later	PICC removed promptly after recognition of malposition and before initiation of hyperosmolar parenteral nutrition

## Discussion

Although malposition of peripherally inserted central catheters (PICCs) into the internal vertebral venous plexus is rare, such malposition carries a substantial risk of severe complications. PICC-related complications, including phlebitis, catheter occlusion, bloodstream infection, and malposition, are known to be associated with catheter tip location [1,2]. In neonatal intensive care unit settings, placement of the catheter tip within the inferior vena cava is recommended when a lower extremity approach is used [1]. The reported incidence of PICC malposition varies widely, ranging from 0.4% to 12.5% [1,2,7,8].

Most previously reported cases of malposition into the paravertebral venous system describe catheter tips presumed to be located in the ascending lumbar vein, primarily based on plain radiographic findings [3]. In contrast, cases in which migration into the internal vertebral venous plexus has been clearly confirmed by imaging are rarely documented [4]. Malposition into paravertebral veins has been associated with a high incidence of severe complications, including fatal outcomes, particularly when hyperosmolar infusions are administered through the catheter [3,9].

The internal vertebral venous plexus is a valveless venous network that communicates extensively with both intracranial and extracranial venous systems and is anatomically closer to the spinal canal than the ascending lumbar vein. Therefore, catheter malposition into this region may theoretically increase the risk of serious complications, such as epidural hematoma, nerve root injury, or intraspinal leakage of infusates. In the present case, computed tomography (CT) imaging allowed direct visualization of the continuous migration pathway from the ascending lumbar vein through the intervertebral vein into the internal vertebral venous plexus. Early recognition of this malposition and prompt catheter removal before the initiation of hyperosmolar infusions, such as parenteral nutrition, likely contributed to the absence of complications.

To the best of our knowledge, reports describing CT-confirmed migration of a PICC from the ascending lumbar vein into the internal vertebral venous plexus in neonates are extremely limited. CT imaging in the present case allowed clear anatomical visualization of the catheter within the internal vertebral venous plexus, whereas in most previously reported cases, catheter position was inferred primarily from radiographic findings. However, similar cases may exist but remain unpublished or unrecognized.

In this case, CT imaging played a crucial role in identifying the precise location of catheter malposition. Chest-abdominal radiography was obtained first for catheter position confirmation; however, before formal interpretation of the radiographic findings was completed, CT imaging was performed primarily for preoperative assessment of the myelomeningocele. As a result, CT imaging incidentally but definitively identified the catheter malposition. Subsequent review of the radiographs demonstrated atypical findings, including unilateral ascent of the catheter along the left side of the vertebral column and medial curvature at approximately the level of the fifth lumbar vertebra. These findings were inconsistent with the expected course of the inferior vena cava and were consistent with previously reported radiographic features of PICC malposition into the ascending lumbar vein [3].

Although plain radiography is the most commonly used modality for catheter tip confirmation, lateral radiographs have also been reported to be useful in selected cases [3,4,9]. Ultrasonography may be helpful for confirming catheter tip position within the inferior vena cava; however, its utility in detecting paravertebral or intraspinal malposition is limited. In neonates with congenital spinal anomalies, body positioning may be restricted, further limiting the feasibility and diagnostic utility of these modalities. CT imaging enables three-dimensional visualization of the catheter trajectory and allows precise anatomical localization. Nevertheless, because CT involves radiation exposure, it should not be considered a first-line modality for routine catheter position confirmation. CT may be justified when conventional imaging findings are inconclusive and potentially dangerous malposition is suspected, in order to prevent catastrophic complications.

There are limited reports examining the relationship between congenital spinal anomalies, such as myelomeningocele, Chiari II malformation, or hydrocephalus, and the risk of PICC malposition. Myelomeningocele and spina bifida are neural tube defects characterized by structural abnormalities of the vertebrae and surrounding tissues, which may influence the anatomy of the paravertebral venous system [5,6]. In the present case, the relatively easy advancement of the catheter into the paravertebral venous system may reflect anatomical variations associated with spinal dysraphism. In addition, the PICC was inserted while the patient was managed in the prone position to avoid pressure on the myelomeningocele lesion. This non-standard positioning may have altered venous flow or catheter trajectory and could have contributed to the unexpected catheter course. However, these mechanisms remain hypothetical, and a causal relationship cannot be established based on a single case.

This case highlights that PICCs placed in neonates can follow unexpected pathways, particularly in patients with underlying congenital anomalies. Accurate confirmation of the catheter tip position before clinical use is essential. When plain radiography demonstrates atypical findings, such as ascent of a lower extremity PICC along the left side of the vertebral column rather than the expected right-sided course of the inferior vena cava, or unusual curvature, clinicians should suspect malposition rather than assuming correct placement. This case underscores the importance of careful imaging interpretation and illustrates the complementary role of CT imaging in selected cases to ensure patient safety.

In our patient, no neurological deterioration or other clinical symptoms were observed, and blood return from the catheter was present, which could potentially lead clinicians to assume correct catheter placement. This case, therefore, highlights that clinical signs of PICC malposition may be absent or non-specific in neonates. Imaging confirmation of catheter tip position is therefore essential before initiating potentially harmful infusions such as hyperosmolar parenteral nutrition.

From a clinical perspective, several practical considerations should be emphasized. First, after lower extremity PICC insertion in neonates, the catheter trajectory should be carefully evaluated on radiography to ensure that it follows the expected course toward the inferior vena cava. Second, ascent of the catheter along the left side of the vertebral column or abnormal medial curvature at the lumbar level should raise suspicion for malposition into the paravertebral venous system. Third, when potentially dangerous malposition cannot be excluded based on radiography alone, additional imaging, such as CT, should be considered. Early recognition of malposition and prompt catheter removal before administration of hyperosmolar infusions are essential to prevent serious complications.

## Conclusions

Malposition of a PICC into the internal vertebral venous plexus is a rare but potentially catastrophic complication in neonates. In this case, computed tomography allowed precise visualization of catheter migration from the ascending lumbar vein into the internal vertebral venous plexus, a finding that could not be definitively established by plain radiography alone. Reports describing CT-confirmed migration of a PICC into the internal vertebral venous plexus in neonates are extremely limited. Clinicians should be aware that PICCs may follow unexpected venous pathways, particularly in neonates with congenital spinal anomalies, and meticulous confirmation of catheter tip position before initiating infusion is essential to prevent serious complications.
